# Rapid, accurate, precise and reproducible ligand–protein binding free energy prediction

**DOI:** 10.1098/rsfs.2020.0007

**Published:** 2020-10-16

**Authors:** Shunzhou Wan, Agastya P. Bhati, Stefan J. Zasada, Peter V. Coveney

**Affiliations:** 1 Centre for Computational Science, Department of Chemistry, University College London, London WC1H 0AJ, UK; 2 Computational Science Laboratory, Institute for Informatics, Faculty of Science, University of Amsterdam, 1098XH Amsterdam, The Netherlands

**Keywords:** binding free energy, ensemble simulation, reproducibility, molecular dynamics

## Abstract

A central quantity of interest in molecular biology and medicine is the free energy of binding of a molecule to a target biomacromolecule. Until recently, the accurate prediction of binding affinity had been widely regarded as out of reach of theoretical methods owing to the lack of reproducibility of the available methods, not to mention their complexity, computational cost and time-consuming procedures. The lack of reproducibility stems primarily from the chaotic nature of classical molecular dynamics (MD) and the associated extreme sensitivity of trajectories to their initial conditions. Here, we review computational approaches for both relative and absolute binding free energy calculations, and illustrate their application to a diverse set of ligands bound to a range of proteins with immediate relevance in a number of medical domains. We focus on ensemble-based methods which are essential in order to compute statistically robust results, including two we have recently developed, namely thermodynamic integration with enhanced sampling and enhanced sampling of MD with an approximation of continuum solvent. Together, these form a set of rapid, accurate, precise and reproducible free energy methods. They can be used in real-world problems such as hit-to-lead and lead optimization stages in drug discovery, and in personalized medicine. These applications show that individual binding affinities equipped with uncertainty quantification may be computed in a few hours on a massive scale given access to suitable high-end computing resources and workflow automation. A high level of accuracy can be achieved using these approaches.

## 1. Introduction

The use of computer models and simulations to understand natural systems is now widespread, encompassing many diverse disciplines in academia as well as industry. One of the major advantages of computational modelling is that it provides insight into underlying molecular interactions and mechanisms, which are often inaccessible experimentally, within the limits of the approximations in the models and the theory concerned. Computer simulations can be performed under conditions where it is difficult or impossible to conduct experiments, for instance, at very high pressures and temperatures. But, beyond the provision of qualitative insight, as our understanding increases one would hope to use these methods to quantitatively predict the outcome of experiments prior to, and indeed even instead of, performing them [1–3]. In this way, computational techniques should reduce time and cost in industrial processes like the discovery of drugs and advanced materials, which take more than 10 years and $2.6 billion for the former [4], and 20 years and perhaps $10 billion for the latter. Due to these potential benefits, computer-based techniques are becoming increasingly popular among researchers from diverse backgrounds, and are adopted as routine techniques by a significant section of the scientific community. The relentless enhancement in the performance of high-end computers is another key factor accounting for the increasing adoption of computer-based methods in science over recent decades.

Given the rapidly growing popularity of computational techniques, it is all the more necessary to ensure that these techniques are reproducible [5,6]. This is essential for such techniques to be relied upon for taking actionable decisions and thereby to become a standard technique applicable in diverse applications, including industrial and clinical contexts. Here, we focus our review on the field of ligand–protein free energy calculation methods based on classical molecular dynamics (MD) simulations and biomolecular systems. A systematic account of the lack of reproducibility of many published results from *in silico* MD and an explanation for their occurrence are provided. Ensemble-based methods are the central focus of our attention since these provide the correct statistical–mechanical way in which to calculate macroscopic quantities such as free energies from microscopic dynamics. They also permit us to perform uncertainty quantification (UQ) in respect of the computed results, and underpin their verification and validation by means of statistically robust procedures. UQ is an established domain in applied mathematics and engineering but has been notably absent *inter alia* from the analysis of computer simulations performed using electronic structure and molecular simulation methods. At this time, we are witnessing unified developments in quantifying uncertainty in computer simulation across a wide range of domains including weather, climate, material, fusion, molecular and biomedical sciences [7–13].

A major goal in drug discovery and personalized medicine is to be able to calculate the free energy of binding of a lead compound or drug with a protein target. That target may be either a generic protein or, in the context of personalized medicine, a sequence-specific variant, reflecting the fact that individuals may respond differently to a given drug based on their genetic makeup. For such calculations to be useful for real-world applications, for example, in drug discovery and clinical decision making, the predictions must be arrived at rapidly, preferably within at most a few hours; manifestly, they should also be accurate and reproducible.

The free energy of binding, also known as the binding affinity, is the single most important initial indicator of drug potency, and the most challenging to predict. It can be determined experimentally by a number of methods, of which measurement of half-maximal inhibitory concentration (IC_50_) provides a semi-quantitative estimate (technically speaking, it is just a ‘proxy’ for the true thermodynamic binding affinity), while biophysical techniques such as isothermal titration calorimetry (ITC) and surface plasmon resonance (SPR) are quantitative, albeit much more time consuming. It should be noted that even the more quantitative measurement methods like ITC are well known to have problems yielding accurate and precise thermodynamic parameters [14]. Indeed, one of the more surprising things about experimental binding affinity data, given their apparent importance in drug discovery, is the extent to which they are reported in widely used databases without any mention of either the measurement method used, or the associated measurement errors.

Alternatively, one may seek to calculate the binding energy theoretically. Here, methods drawn from computational chemistry offer a route forward; these are primarily based on *in silico* MD, for which several approaches to determining the free energy are possible. Methods that rapidly predict binding affinities are preferable in the context of personalized medicine and drug discovery but, as we shall see, there is a trade-off between computational cost, accuracy and precision. As a consequence of the conflation of experimental methods and their unknown error distributions referred to in the preceding paragraph, these computational approaches are hindered in a number of ways. Nonetheless, by advancing the accuracy and precision of theoretical methods one may expect to encourage more care to be taken in reporting similar attributes of experimental binding energies.

The widespread use of molecular simulation for free energy calculations, especially in the field of pharmaceutical drug development in the last few years, is now placing a premium on our ability to deliver *actionable* predictions to academic, industrial and clinical communities. For knowledge to be actionable in the current context means that the predictions are accurate, precise and reproducible, and are made on time scales that are sufficiently rapid to be used in a decision-making context. The most familiar example of actionable predictions arises in weather forecasting, as well as climate science, where ensemble-based methods play a central role [15,16]. People wish to know tomorrow's weather today, not tomorrow let alone in three months' time. Having a reliable probabilistic prediction prior to an event taking place is extremely valuable and arguably represents the apotheosis of the scientific method in action. There is a growing awareness of the importance of making actionable predictions for a range of real-world problems, reflected in recent literature covering a range of disciplines including natural disasters, climate change and medicine [17–20]. This review aims in part to enhance awareness of the issue in computational chemistry and molecular simulation in particular.

## 2. Dynamical systems, ergodic theory and statistical mechanics

All the free energy methods we shall describe are based on the use of classical MD. The dynamical observables, *G*, are calculated as macroscopic averages, which are given by ensemble averages, denoted 〈*G*〉*_t_*. Thus

$${\left\langle G\right\rangle }_{t}=\int G(x)\rho_{t}(x)\text{d}\mu ,$$

where *x* denotes the 6*N* phase space variables, μ is the invariant measure associated with it and *N* is the number of particles in the system. The ergodic theorem is commonly invoked within the domain; it states that, in the long time limit, a single trajectory generates a time average of a dynamical observable, 〈*G*〉*_t_*, that is identical to its ensemble average 〈*G*〉_eq_:

$${\left\langle G\right\rangle }_{\mathrm{eq}}=\underset{t\to \infty }{\lim}{\left\langle G\right\rangle }_{t}=\underset{t\to \infty }{\lim}\int G(x)\rho_{t}(x)\text{d}\mu =\int G(x)\rho_{e}(x)\text{d}\mu ,$$

where $\rho_{t}$ and $\rho_{e}$ are respectively the (6*N* + 1) dimensional time-dependent and equilibrium probability distribution functions defined on the phase space. $\rho_{t}$ satisfies the Liouville equation [21]. A time-independent state is asymptotically approached if the dynamical system possesses an equilibrium state such that

$$\underset{t\to \infty }{\lim}\rho_{t}=\rho_{e}.$$

To be ergodic, a system must pass through every possible point in phase space on the energy shell (in the microcanonical ensemble). The probabilistic description of the dynamical behaviour should be invoked; although usually stated as being equivalent to the deterministic Newtonian, trajectory based, formulation of classical mechanics its conceptual basis is quite distinct and admits the inclusion of statistical mechanical concepts which are lacking in the trajectory-based approach [21]. The problems surrounding the reproducibility of the method are rooted in the instability of the MD trajectories that underpin it, rendering them increasingly inaccurate as time evolves [21]. Perhaps surprisingly, this fundamental issue is something that has been frequently overlooked in the literature. It is perhaps not well known [21–27] that many complex systems, including all those to which statistical mechanics is applied in order to calculate equilibrium states and their properties, exhibit extreme sensitivity to initial conditions. Briefly, in the ergodic hierarchy of dynamical systems, systems which approach and reach equilibrium must be at least mixing [21]. Neighbouring trajectories—the solutions of the Newtonian equations of motion—in such systems, no matter how close they are initially in phase space, diverge exponentially fast with the passage of time [21]. Under such circumstances, the notion that we can, even in principle, specify by some experimental procedure the initial conditions for the time integration of the dynamics is undermined. Instead, we are obliged to formulate the approach to equilibrium in probabilistic terms [21,28,29], which is to say in mathematical language based on measure (also known as Lebesgue) theory. That theory holds almost everywhere, except possibly for a set of zero measure. But everything we can compute is based on a very small subset of the computable numbers, the IEEE floating-point numbers, both of which are sets of zero measure, the latter being a very small subset of the rational numbers (they are dyadic numbers, that is numbers with power of two denominators). Representing behaviour of dynamical systems using floating-point numbers may lead to the omission of a considerable amount of the structure of a dynamical system as has recently been pointed out [30]. Therefore, there is the likelihood that the calculations performed on modern digital computers, using the IEEE floating-point numbers, may not always correctly describe the probabilistic properties of chaotic dynamical systems [30]. The ramifications of these limitations remain to be fully understood but our expectation is that they may typically contribute systematic errors of order unity to the expectation values computed on digital computers. Given that, for the computation of free energies in this way, we are calculating expectation values which are estimated by ‘sampling’ from what one hopes to be the true probability distribution, this sampling must be performed in a manner that is sensitive to all possible sources of uncertainty.

**Figure 1.**
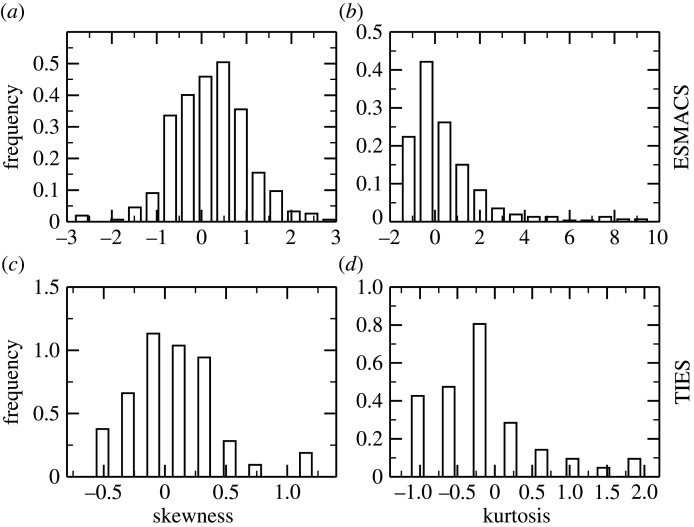
Non-Gaussian properties of equilibrium distributions from ensemble MD simulations. The Fisher–Pearson coefficient of skewness (*a*,*c*) and the Fisher kurtosis (*b*,*d*) for distributions of predicted binding free energies using ESMACS approach (*a*,*b*) and free energy differences from TIES calculations (*c*,*d*). The ESMACS studies consist of approximately 400 ligand–protein complexes, and the TIES studies include alchemical mutations of 50 pairs of ligands. The distribution of calculated binding free energies for each ligand comprises 25 independent replicas within an ESMACS simulation; the distribution of binding free energy differences for each pair of ligands comprises 20 or 40 replicas in a TIES simulation. The distributions are skewed both left (negative values of the skewness) and right (positive values). For the distributions with kurtoses less than 0, the distributions have short and thinner tails, and are more flat-topped than the normal distribution would predict. For the cases with kurtoses greater than 0, the distributions have a heavy tail, usually at the right for ESMACS (*b*) because the binding free energy has a lower bound (on the nanomolar or picomolar level). The large kurtosis values in these studies indicate that such simulations produce significantly more ‘outliers’ than one would anticipate were the statistics to conform to a normal distribution.

Extensive studies we have performed in recent years confirm that MD models indeed exhibit sensitivity to initial conditions [24–26,31]. From our investigations, we find that the frequency distribution of observables as it emerges from the members of an ensemble exhibits deviations from the standard Gaussian profile anticipated on the basis that the variables are independent of one another, as one assumes in conventional statistics [32]. Instead, we find that the distributions have a skewness associated with them, the asymmetry favouring the occurrence of values of the observable higher than the mean (figure 1*a*,*c*). The majority of the distributions have positive kurtosis, meaning they are heavy-tailed relative to a normal distribution (figure 1*b*,*d*). This is at first sight unexpected—it has not previously been reported in the context of classical MD—until it is recognized that these systems all display chaotic behaviour as well as long-range interactions; the underlying nonlinearities in the dynamics are what accounts for both the presence of chaos and non-Gaussian statistics. In addition, the distribution of experimental binding free energies might not be Gaussian. The phenomenon is well known in turbulence: there it is caused by very long-range hydrodynamic interactions mediated by energy dissipation. The reason for the presence of non-normal statistics in biomolecular systems at equilibrium comes from the fact that here too we are dealing with the infinite range interactions mediated by Coulomb forces. The dissipation of energy within the system causes long-range correlations to be set up, which manifest themselves in the non-Gaussian nature of the statistics, leading to the more frequent occurrence of outliers than otherwise would be expected. Graphs of theoretical versus experimental data will not produce ideal linear plots in which all points converge closely on the 45° line. Instead, those working in this domain should expect to observe many more so-called outliers as a consequence of the natural behaviour of these systems. Although no explicit mention is made of the fact by the authors, Knapp *et al*. [33] is replete with figures displaying skewed (i.e. non-normal) distributions of geometrical quantities emanating from large ensembles of protein MD trajectory data. Statistical tools like bootstrapping and linear regression do not assume any underlying distribution. Therefore, in principle, they should be applicable to non-normal observables, such as MD-based free energies. However, their quantitative reliability is debatable for non-normal distributions in the absence of sufficient samples [34,35]. This further adds to the necessity of performing ensemble simulations for MD-based methods. Additionally, estimators like median-of-means allow heavy tails, are robust to outliers in the data and hence may be used to estimate means and variances for such distributions. As soon as this behaviour is apprehended, one understands why the outcome of single simulations is in general not reproducible, and may lead to false-positive conclusions [33]. It has an anecdotal quality. The next person who studies the same system is likely to obtain very different results (as indeed one observes in the literature when, for example, one paper reports the observation of a conformational change while another does not, from the very same system [21]).

Our findings serve to underscore that we need a statistical theory of MD simulation, in the same way that there is an established statistical theory of turbulence [36]. Individual trajectories are not robust indicators of molecular behaviour, and owing to chaos long duration single trajectories lack accuracy, but we expect statistical averages over such trajectories to be knowable to high precision. In this way, we can distinguish random from systematic errors, the former arising due to the chaotic nature of the dynamics, the latter to errors in things like the force field parametrizations employed. Without first correctly handling the stochastic errors, it is not possible to assess correctly the nature/size of the systematic errors. Our recent work [30] shows, however, that even statistical averaging can produce hitherto unexpected systematic errors, caused by the fact that the IEEE floating-point numbers are a poor representation of the real numbers. We have shown this recently for the case of simple dynamical systems for which the equilibrium probability distribution is known exactly [30]. For the systems studied by practitioners of MD and turbulence, such probability distributions are not known and must be assessed by sampling methods. Based on our studies of very simple ergodic systems, the errors accruing from the use of floating-point numbers are, in specific cases, likely to be catastrophically large; in others they are more insidious, in that simulation results may seem correct but will contain errors of order unity. In the most favourable cases they will contain errors that are small, but nonetheless much larger than machine precision.

Putting those fundamental limitations of floating-point numbers aside, the calculation of observable stochastic quantities—which are essentially expectation values—proceeds through an ensemble approach, in which a set of independent MD simulations, referred to as ‘replicas’ in statistical mechanics, is performed and averaged over both time and the members of the ensemble (see details in the following subsection). The key feature of ensemble simulation is the use of ensemble and time averaging [21]. The criterion for ensuring convergence of the ensemble average is to establish the number *N* of replicas required such that using *N* + 1 of them makes no difference to the expectation values calculated. This is very different from calculations using a few repeats of a single MD simulation [37,38] including replica exchange [39], which do not permit reliable estimation of errors. Here, we describe our procedures for performing rapid and reproducible binding affinity calculations, and present the results for a number of ligand–protein cases. The approach is scalable: the throughput of results depends primarily on the size and speed of the available computer. Recent work [40] on both the calculation methods and the cyberinfrastructure environment could transform drug design by supporting accurate and rapid calculations of how strongly compounds bind to target molecules, a high-performance application that can scale to the largest supercomputers available in the world today and well beyond [41].

### 2.1. Making molecular dynamics simulations reproducible

Scientific results are by definition supposed to be reproducible, that is they should be independent of who conducts the study. Within the scientific community, there is a somewhat confused terminology addressing the confirmation of the correct measurement or phenomena, which includes such terms as reproducibility, replicability and repeatability [6,42]. We use the term ‘reproducibility’ in the context of this review to refer to the ability of a method, be it experimental or theoretical, to yield the same results when repeated *by oneself or others*, *with or without variation in its implementation* including the software and the hardware employed. Such reproducibility, which necessarily has a statistical nature, is essential if a technique is to be used, for example, in a medical context to treat human beings, where there are stringent regulatory requirements. But it is also fundamental to the development of reliable scientific methodologies in much wider contexts [43].

A method cannot be reliable if it does not yield the same result when performed by oneself, let alone others. Indeed, the lack of reproducible results in the published literature is a widespread concern in the scientific community [6]. Measurements are not perfect. They always contain errors. So too for a theoretical result. The issue was recently highlighted by a survey conducted by *Nature* which found that more than 70% of researchers failed to reproduce another researcher's results, while more than half were unable to reproduce their own [44]. In the case of experiments, non-reproducible results can be an artefact of factors ranging from the incorrect use of chemicals, an insufficient number of samples, fluctuations in the environment and variations in the experimental setup, to data dredging and *a posteriori* hypothesis generation, not to mention conclusions influenced by conformational bias, conflicts of interest, selective reporting or, worst of all, misconduct. In the case of computer-based methods, the reasons may also reside in the theory or the model used, the extent of convergence of the calculations, the reliability of the software, the adequacy of the floating-point representation of the real numbers [30] and so on [27].

In this review, we focus on the convergence, reproducibility and reliability of observable properties obtained from MD simulations. Although it was recognized more than two decades ago that one-off classical MD simulations do not generate consistent protein conformations [45,46], systematic investigation as to how to make these calculations reproducible has not been performed until recently. This is a reflection of the history of how ensemble methods became adopted in weather and climate forecasting: pioneered some 25 years ago, their initial introduction encountered considerable resistance from established workers in the domain but they are now the standard method by means of which probabilistic weather forecasts are made on a daily basis [16]. In the molecular simulation domain, one notices similar reluctance to embrace the probabilistic approach. Until now, one-off simulations remain a very common way of performing MD studies (see ‘Application of free energy calculations' section below).

More specifically in the field of MD-based free energy calculations, the variation in the calculated free energies based on independent simulations was investigated systematically by Sadiq *et al.* [24] and by Genheden & Ryde [47] using MMPBSA and MMGBSA methods, respectively. The estimated free energies from two independent MMPBSA calculations of the same molecular system can vary by more than 10 kcal mol^−1^ in smaller molecule–protein complexes [26,31,47], and by up to 43 kcal mol^−1^ in larger and/or more flexible ligands bound to a protein such as the peptide–MHC (major histocompatibility complex) systems [25]. The underlying reason for such variations between independent MD simulations is due to the extreme sensitivity of a dynamical system to its initial conditions [21,48].

Although these studies [21,24,45–47] employed various methods to calculate diverse observable quantities, the common conclusion was drawn that multiple short MD simulations provide substantially better sampling than a single long MD simulation. This does not invalidate the ergodic theorem, it merely indicates that the time scales over which MD simulations are run are nowhere near long enough to fulfil its requirements. It should be noted that these studies investigated systems which were at thermodynamic equilibrium; for behaviour that requires long time scales to happen (such as conformational changes), including also the important case of non-equilibrium systems, we must use ensembles for both long and short simulations. Further investigation has been undertaken in the last few years, which has led to approaches such as enhanced sampling of MD with an approximation of continuum solvent (ESMACS) [25], velocity-induced independent trajectories (VIIT), solvation-induced independent trajectories (SIIT), conformation, rotation and protonation-induced independent trajectories (CRPIIT) [49] and methods-induced independent trajectories (MIIT) [50]. Similarly, in the case of alchemical methods, Lawrenz *et al.* [38] introduced a method called independent trajectory thermodynamic integration (IT-TI) which employs multiple, independent TI calculations and yields more accurate free energy changes. More recently Bhati *et al*. [51] published a method called thermodynamic integration with enhanced sampling (TIES) which employs an ensemble of independent MD simulations in combination with the concept of stochastic integration to yield accurate and precise free energy predictions. While most of the aforementioned approaches only make use of multiple separate simulations and/or independent trajectories but do not systematically assess the statistical or the statistical mechanical significance, ESMACS and TIES exploit the statistical mechanical concept of ensembles and the connection to ergodic theory to quantify uncertainty and obtain reproducible results from MD simulations in a systematic and theoretically well-grounded manner [21].

### 2.2. Sources of error in classical molecular dynamics

As noted above, there are two sources of error accruing in MD simulations, due to systematic and random sources. The systematic errors originate in things like the imperfect methods, models and calibration of simulations. Biases of protein force fields towards different secondary structure types [52], for example, will consistently populate either helical or sheet-like structures from independent simulations. When the cause of such systematic errors can be identified, it can be reduced or even eliminated, as shown in recent simulations with state-of-the-art force fields [53].

Random variation, also called system noise or stochastic error, on the other hand, has a different origin. It is caused by the chaotic nature of classical MD. Given the sensitivity of Newtonian dynamics to initial conditions, two independent MD simulations will sample the microscopic states with different probabilities no matter how close the initial conditions of the simulations [21]. The difference produced by two simulations introduces a level of variation which can be larger than the quantity of interest, making the results practically useless.

In order to get a full grip on uncertainty in MD simulations, one needs to be able to identify the systematic and random components contributing to the errors. We would like to assess the variation in results arising from the inaccuracy inherent in the molecular models including the choice of force field. This has not been convincingly addressed to date, since for the most part the UQ due to the intrinsic random error in the simulation trajectories is only now being nailed down. The overall quality of a computer-based simulation study can be assessed by a relevant set of verification, validation and uncertainty quantification (VVUQ) methodologies and associated tools [54,55]. Validation is about comparing the results of our models with experiment (or high-quality reference benchmark/theoretical results) while verification is concerned with ensuring that the quantities we are calculating from the algorithms and software are themselves being computed correctly. UQ reports the error in the calculations. It is timely to address these issues now and one purpose of this review is to encourage such investigations (see for example https://www.vecma.eu/). One way to approach these issues is through sensitivity analysis in the first instance—which term or terms in a force field or other choices made, such as cut-off distances, size of simulation cell, etc. lead to the greatest sensitivity, for example in the calculation of the free energy of binding of a ligand to a protein?

### 2.3. Ensemble averaging

In order to address the aforementioned problems, it is necessary to perform ensemble simulation, requiring a break-away from the traditional practice of performing one-off MD simulations. The practice of one-off simulations, based on an *ad hoc* appeal to the ergodic theorem discussed earlier, has been at the basis of most publications in the field since the dawn of MD which was originally introduced by Alder & Wainwright in the late 1950s [56]. We focus here primarily on free energy methods which have been used for decades to study the binding affinities of ligands to their target proteins [57].

The results from ensemble simulations are designed by construction to be accurate, precise and reproducible [21,24–26,47,50,51,58–61]. It should be noted that the term ‘accuracy’ refers to the closeness of the results to the corresponding experimental values and it is largely dependent on the limitations of the force field employed. In addition, given the vast number of nodes, cores and accelerators on modern high-performance computers and available automated workflows (see the ‘Distributed computing approaches to enhance sampling’ section below), all of the replicas can be run in parallel and hence an ensemble simulation can be run in the same wallclock time as needed for computing a single replica [25,51]. This leads to rapid predictions informed by high-quality error estimates, which is essential for free energy methods to have an impact beyond an academic setting. The appropriate number of replicas and the duration of the simulation are parameters dependent on the system under study and the calculational method selected; they depend on the extent of stochasticity, that is the fluctuations, within individual stages in a calculation comprised of multiple steps, and the level of precision desired, although general guidelines are available [21,25,26,51,61]. Thus, for example, within a relative binding free energy calculation using a thermodynamic cycle, the alchemical leg for the ligand–protein complex requires more replicas than that for the ligands to realize the same level of precision [61]. The errors in each individual step decrease as the inverse of the square root of the number of replicas, $1/\sqrt{N_{\mathrm{replicas}}}$. Varying numbers of replicas are required to achieve a desired level of precision; for example, of order 25 replicas are typically required for ESMACS studies [25,26], 5 replicas for each *λ* window within a TIES relative binding free energy calculation [51], a combination of 5 and 10 replicas during the multiple steps required to compute absolute alchemical binding free energies [61] and as many as 40 replicas for some graphene-related MD studies [62]. Many ensemble simulations only vary the velocities in the initial conditions of the replicas [24,25,51,61]; in some cases variations in the initial spatial coordinates are also required [63].

Considerable effort has been invested in the development of so-called ‘enhanced sampling protocols' in order to improve phase space sampling [64–66], including metadynamics, a method for accelerating rare events in simulated systems [67]. Among these, the most popular in the case of biomolecular simulation is the Hamiltonian-replica exchange (H-REMD) [68] and its variants—replica exchange with solute tempering (REST2) [69] and FEP/REST [70]—which run multiple concurrent (parallel) simulations and occasionally swap information between them to improve sampling. A molecular system subjected to these parallel simulations has a common configuration space; the simulations sample the microscopic states with different probabilities because of the differences in their Hamiltonians. For a given set of simulation samples, different free energy estimators can be applied with varying reported accuracies and precisions [71]. One of the free energy estimators is called the multistate Bennett acceptance ratio (MBAR) [72] which has become increasingly popular of late. MBAR makes use of all microscopic states from all of the replica exchange simulations, by reweighting them to the target Hamiltonian.

Free energy calculations had rarely been used seriously in drug development projects until recently when Schrödinger Inc. released their ‘FEP+’ simulation software for relative free energy calculations [73]. With the improved technology and the availability of graphical processing units (GPUs), FEP+ has made a significant impact in the pharmaceutical industry within its domain of applicability [74]. FEP+ is employed in a shrink-wrapped and very easy to use manner, being wholly proprietary and directed at commercial users. Unfortunately, it promotes the uncritical use of one-off simulations. Merck recently published a large-scale study using FEP+ and discussed several challenges in its application within the drug discovery process [75]. However, the authors did not perform ensemble simulations to confirm the robustness and reproducibility of their findings which therefore have only a provisional status. In an attempt to gain a handle on errors in these calculations, FEP+ recommends the use of closed thermodynamic cycles of transformations performed by one-off simulations in order to detect hysteresis and, indirectly, to assess convergence. While this method can indicate that convergence has not been reached, a small error reported in cycle closure convergence does not guarantee convergence. This is because, while a hysteresis value of 0 from such a closed cycle is a necessary condition, it is by no means sufficient. Hysteresis certainly merits close attention, as indeed we do within TIES, for example, when comparing results from simulations running several ‘forwards’ and ‘backwards’ transitions [76]. In such cases, this amounts to a form of ensemble simulation in which replicas with different initial and final states are used.

It is often claimed that the implementation of an enhanced sampling protocol such as REST2 [69] and the use of the free energy estimator MBAR [72] can overcome the problem of non-reproducible results. This is not the case. Application of REST2 may make a ligand drift away from its stable binding position, and lead to deteriorated free energy predictions [77]. In recent work, we performed free energy calculations using FEP+ [73] which *de facto* implements REST2 and MBAR. Up to 3.9 kcal mol^−1^ variations were observed from 30 independent simulations, much larger than the MBAR errors reported for individual FEP+ calculations [78]. Other studies have also found that bootstrap analyses from repeated simulations provided a more realistic uncertainty estimate than MBAR [79]. It is clear that such ‘enhanced sampling’ methods are not an alternative to the use of ensemble simulations [61]: they too must be ensemble averaged [61,77,78].

## 3. Common methods for free energy calculations

Molecular recognition [80] is central for many physical, chemical and biological processes. Accurate prediction of the binding affinity of a guest molecule with its host is an important goal in host–guest chemistry and has an essential role in biomolecular signalling and pathways. A guest is often a compound with low molecular weight, while a host is usually a larger molecule which encompasses the guest. Guests are the so-called ligands we consider here, which are molecules that bind reversibly and specifically to a biomacromolecule (a protein in the context of this paper) and alter the latter's activity. MD simulation provides a tool to collect microstates of the biomolecule of interest, and has been applied to get the macroscopic thermodynamic properties, such as the free energies, from the ensemble of microstates.

### 3.1. Free energy of binding

The binding affinity is the change in the free energy associated with a binding process. The magnitude of the binding affinity is a measure of how strong the interaction is between the ligand and the protein, and hence it is often directly related to the potency of the ligand. Therefore, its measurement is of importance in the fields of drug design and personalized medicine. It can be used as a virtual screening tool in drug design or as a clinical tool to tailor a patient's medication based on his/her genetic makeup. Computer-aided drug design (CADD) is an extremely active field of research [81]. In addition, rapid and accurate binding affinity predictions can be useful in health-related applications like the design of medicines with reduced side-effects and drug resistance [82]. Thus, the use of *in silico* techniques to predict binding affinities has grown immensely in the last few decades [83].

Reliable binding affinity predictions need to be made on time scales shorter than experimental ones in order to have a real impact in drug design and personalized medicine [82]. Therefore, the time to solution is another important factor influencing the applicability of computational methods in real-world scenarios. This is especially crucial when considering the development of a typical prescription drug which, as noted, often takes around a decade (and costing $2–3 billion) to get to market [4]. The ensemble approaches described above fulfil these requirements given the availability of sufficient computing resources (for more details see the ‘Application of free energy calculations’ section).

### 3.2. Methods for free energy calculation

The methods with most potential, in order of increasing level of molecular resolution, are listed below. A higher level of resolution ought, in principle, to lead to higher accuracy, although this is not necessarily true because of the quality of the theory employed and the way in which the calculations are implemented [27].

(i) ‘Informatics’ based approaches which are usually the output of docking studies in combination with so-called ‘machine learning’ [84–87];
(ii) linear interaction energy (LIE) methods [88];
(iii) molecular mechanics Poisson–Boltzmann surface area (MMPBSA) and molecular mechanics generalized Born surface area (MMGBSA) methods [89] based on invoking a continuum approximation for the aqueous solvent to approximate, e.g. electrostatic interactions following all-atom MD simulations; and
(iv) alchemical methods including thermodynamic integration (TI) and free energy perturbation (FEP).

Machine learning (ML) is currently gaining a lot of traction within the pharmaceutical industry. The current approach is to seek to invoke ML to generate candidate compounds and to rank congeneric compounds [90]. There are now many start-ups and small companies that offer such ‘AI’ (artificial intelligence) based approaches to drug discovery; this is being done to generate lots of candidate compounds, both virtual and real. The predictive performance of ML methods for binding affinities, however, is sensitive to the quality of the ligand–protein structures which are usually generated using docking methods. There are claims that ML can achieve ‘chemical accuracy’, meaning ±1 kcal mol^−1^ in energy predictions. Indeed, it has been shown that, when the most relevant high-quality data are used for training, ML algorithms can generate accurate binding affinity predictions [91,92]. This is still hotly contested in real-world situations due to a number of shortcomings of such approaches [1,2,93]. These arise from some obvious built-in assumptions of all ML algorithms. The key one is the assumption that relationships between points in ML data space are smooth (continuously differentiable), so they interpolate smoothly between gaps in the state space. This may or may not be valid, depending on each and every case under study; when invalid, however, its predictions will fail badly. Thus, for example, when the free energy changes more or less discontinuously with molecular structure, as it does for the case of free energy cliffs [94], there is no way such an ML algorithm will in general be able to spot such phenomena. It could only do so if the coverage of the state space were exceptionally dense, implying that the data upon which it has been trained would need to be enormous. A related generic problem is the well known ‘curse of dimensionality’: for a state space of dimensionality *N*, the required quantity of training data grows as an exponential function of *N*, so that there is no chance of acquiring sufficient data to get close to densely populating the state space of a complex system with representative examples. ML is, at root, nothing more than glorified curve fitting; and equipped with so many thousands of adjustable fitting parameters, it is no wonder that it may appear to fit the data it has been trained upon well. In general, however, this leads to overfitting, meaning that it then often fails spectacularly but unexpectedly when asked to make predictions for previously unseen data [1,2]. Lacking any significant explanatory power, it is hard to figure out what has caused the poor performance.

More accurate experimental and computational chemistry studies are needed to provide correct binding poses and binding affinities in conjunction with it. Combinations of ML and MD are currently being used, for example, to search for appropriate evolution of MD simulations through various ‘on-the-fly learning’ processes [95,96]. Because of the high compute intensity of MD calculations, ML is being increasingly used as a ‘surrogate’ in order to replace that expense with something less costly and time consuming. It is hoped that combinations of ML and MD [97] will enable the virtual screening of colossal numbers of compounds, and to focus only on a small subset of those virtual compounds with more computationally expensive free energy calculations. This, in turn, should ultimately lead to much more limited effort and cost expended on the actual synthesis and testing of candidate compounds for subsequent drug development.

The LIE approaches usually generate worse relative binding affinity rankings and considerably larger uncertainties than MMPBSA and MMGBSA [98]. In addition, the scaling factors for the electrostatic and the van der Waals interactions in LIE approaches are still a matter of discussion, and the quality of predictions from the approaches is frequently reported to be system-dependent [98].

Ensemble-based approaches centred on (iii) MMPBSA/MMGBSA and (iv) alchemical methods are the focus of our attention in this review. Although MMPBSA/MMGBSA means many different things in the literature, when we refer to it here we mean the full determination of the free energy of binding from either a one-, two- or three-trajectory method: it includes both the configurational entropy and the association free energy [26,99], and—where appropriate—the adaptation energy [25,59,60]. It may be invoked, in principle, to any inhibitor–protein complex, although caution should be applied when truly diverse datasets are handled [100,101]. ESMACS is the name we give to this protocol when it is run in an ensemble-based form, with all these options available to select from. Recent ESMACS publications [25,59,60,100,102], for example, investigate drug-like small molecules bound to therapeutic targets, including G protein-coupled receptors (GPCR), the most frequently exploited drug target class, as well as biological substrates (9-mer peptides) bound to the major histocompatibility complex (the p-MHC system) of central importance in immunology. The peptides in the latter are much larger than common small-molecule drugs, and have widely varying structures and electrostatic charges. The methods have also been used to study protein–protein interactions [103,104]. ESMACS is thus well suited for use in the initial hit-to-lead activities within drug discovery.

The alchemical methods have a more restricted domain of validity: they are applicable mainly to estimating small relative free energy changes for structures (drugs or proteins) which involve relatively minor (perturbative) variations. As such, the methods are most relevant to lead optimization following the identification of promising lead compounds. When changes in the net charge arise, TI and FEP methods encounter specific difficulties owing to major adjustments in long-range electrostatic interactions [105]; charge correction approaches are required to take into account the artefacts of the electrostatic potential energy introduced by the finite size effects [106]. A recent paper by Wang *et al.* [73] employing FEP has attracted significant attention, as it purports to provide a reliable route to the prediction of binding free energies. However, it has the same restricted scope as it seeks to compute free energy differences between similar ligands bound to a protein. The approach advocated has been to run single simulations, without paying any attention to the stochastic nature of the quantities calculated. A recent study furnishes an estimate of the reproducibility of TIES and provides a reliable method for UQ for both relative and absolute binding free energy (ABFE) calculations using alchemical methods [61,78]. While equilibrium simulations are commonly implemented in alchemical methods, there are also non-equilibrium approaches which can generate comparable results with these obtained from equilibrium simulations [107–111]. The accuracy of such approaches is quantified by comparing the predictions with experimental data which, as previously discussed, all too often have no associated errors. Compounding this, there is frequently no reporting of the variations arising from the predictions provided by the calculational method, leaving the uncertainty associated with the protocol largely unquantified.

While ESMACS (and LIE) is an ABFE method and TIES/FEP+ are relative free energy methods, there exists an alchemical ABFE method which can be used to estimate binding affinities, which we now describe. It is the equivalent of TIES/FEP+ when one of the drugs involved is replaced by nothing, in both bound and unbound states. The calculation method is called double annihilation, first proposed three decades ago [112]. A series of nonphysical steps are involved in the calculation; the free energy changes for each step are calculated by a combination of alchemical and analytical methods [61,79,112]. The processes of decoupling/coupling the ligands from/to the environment involve large changes in phase space, of which the calculated free energy changes exhibit large fluctuations. Owing to its extreme compute intensity and intrinsically large uncertainties, the method has until recently not been applied to pharmacologically relevant proteins in any significant manner. Ensemble approaches render ABFE much more reliable, and reveal that in this compute-intensive multistep calculation, the various steps require different ensemble sizes to attain the same high level of precision [61]. The ABFE calculation is by far the most expensive of the methods we discuss here. Compared with the pair-wise comparison of the relative binding free energy calculations, the advantage of ABFE approaches is that their results can serve as a reusable library to which calculated ABFE results for other ligands can be compared and added.

The use of these approaches is not mutually exclusive but indeed can be even more powerful when performed in tandem. For example, a combination of endpoint and alchemical methods has been used to accurately predict protein–ligand interactions for a membrane transporter [113]. In the currently ongoing COVID-19 pandemic, the computer-aided drug discovery market has experienced a boost, in which the aforementioned approaches have been extensively applied, separately or jointly, to find novel drug candidates and to reposition existing drugs. We ourselves are currently participating in a large scale collaboration in which ML, docking, endpoint and alchemical approaches are applied interactively to find promising drug candidates from data consisting of billions of compounds. The most attractive drug candidates are subsequently being studied experimentally, with some under consideration for inclusion in possible clinical trials.

## 4. Ensemble-based simulation approaches

Over the past 20 years, all these methods have been subjected to substantial criticism for a wide range of reasons, mainly due to their lack of accuracy and reproducibility, and in the case of (iv) their long turnaround time. Two distinct but related problems contribute to the issue: conformational exploration and precise sampling. The usage of ensemble approaches is increasingly widespread within a broad range of MD studies, for sampling ‘rare events’ including protein folding and ligand binding kinetics using ensemble dynamics [114], weighted ensemble [115] and splitting methods [116], for predictions of residence times using steered MD [117] and random acceleration molecular dynamics (RAMD) simulations [118], and extending now from all-atom to multiscale and coarse-grained studies [62,119,120].

### 4.1. Ensemble-based conformational exploration

Many biological processes occur on time scales which are difficult, if actually possible, to access by atomistic MD simulations. Such processes usually go through a complicated free energy profile which can be simplified into local minima and transition states. The former normally trap a system for very long times, while the latter can only be accessed rarely and transitorily [9]. Transition path sampling [55] is one common approach to investigate the transition paths connecting different minima, in which accelerated MD approaches such as metadynamics [67], steered MD [117] and high-temperature simulations are first used to construct an overall free energy landscape, followed by ensemble simulations from putative transition states to generate a transition path containing information on the mechanism and kinetics of the process [121]. The ensemble simulations consist of many relatively short runs sampling regions between different minima but do not need to spend a long time in any specific minimum. A converged free energy landscape can then be reconstructed once all of the regions have been fully explored [121], if the weighting factors can be correctly assigned to each conformation [61]. A widely used approach is to construct a Markov state model (MSM) [122,123] for the description of biological processes such as ligand binding and protein folding. Large scale ensemble simulation needs to be run to adequately sample the entire configurational space, which consists of a vast number of individual MD simulations.

Among ensemble simulation approaches used for studying long time scale events by accessing transition states are ensemble dynamics [114], weighted ensemble methods [115] and multilevel splitting along with their variants [116]. These methods differ from the ones described above in that they do not involve any external force or biasing potential; nor do they involve heating the system of interest. Rather they exploit the fact that MD simulations, being chaotic, are extremely sensitive to their starting conditions and enhance sampling of otherwise inaccessible states by running large numbers of short independent MD simulations varying only in their starting conformations. The ensemble dynamics method has been successfully used to study protein folding of a large number of systems in the last couple of decades [124]. It involves replacing a single long simulation by an ensemble of shorter simulations with a cumulative simulation time of up to microseconds. The fraction of simulations capturing folding or a conformational change of interest is used to infer the probability of such events. The weighted ensemble method involves partitioning the phase space into several regions and initiating an equal number of concurrent ‘walkers’ in each of them [115]. The regions are maintained at equally populated levels by adjusting the number of walkers in each after regular intervals. This permits sampling of rare events that would otherwise get underpopulated with walkers and hence sampled less. The basic idea behind the multilevel splitting method is to discard trajectories that drift away from the region of interest in the conformational space while focusing on those that get closer to it [116]. To this end, a ‘reaction coordinate’ is defined that is used to monitor the progress of a trajectory and to measure its closeness to the desired rare event. This allows one to dedicate the majority of computational effort to sampling the region of interest instead of the initially much vaster phase space. It should be pointed out, however, that although all these methods use ensemble simulations, they do not do so to ensure reproducibility, but only as a means to capture and observe interesting transitions. Thus, the application of UQ in these contexts still remains wide open.

#### 4.1.1. Ensemble-based docking

Ensemble methods have been used in docking studies to accommodate the flexibility of target proteins, in which an ensemble of structures can be generated from explicitly solvated MD simulations, against which the screening of ligands is performed. It has been demonstrated that the approach generates a set of top hits, some of them ranked very poorly if only crystal structure data are used [125]. Our own work [126] also showed that ensemble-based docking is required to explain the preference of gatekeeper mutant EGFR (epidermal growth factor receptor) binding with gefitinib, a targeted anti-cancer drug, rather than ATP. To improve ligand–protein binding affinity predictions, multiple independent MD simulations have been applied within the LIE [127] approach. Ensemble docking approaches have been employed recently to identify small molecules which may disrupt host–virus interactions at an entry point for infection with the SARS-CoV-2 [128].

#### 4.1.2. Ensemble-based peptide and protein folding

Since the pioneering study by Duan & Kollman [129] two decades ago, significant advances have been made in the field of peptide folding studies [130], thanks to the rapid development of simulation approaches, the availability of powerful supercomputers, and the improvement of the force fields. Using vanilla all-atom MD, the lengths of simulations for peptide (un)folding have reached the time scales in the range of microseconds to milliseconds [130]. The Shaw group has applied long time scale simulations, up to 1 ms, correctly folding 12 structurally diverse small proteins to their experimentally determined structures using Anton [131], a special-purpose MD supercomputer. Except (un)folding in solvent, the process of peptide binding, folding and partitioning into lipid bilayers has been successfully captured using high-temperature MD simulations [132]. Because of the time scales required for the simulation of peptide folding/unfolding, it is not surprising that many of these standard simulations use a single trajectory approach, lack accuracy and reliable error estimates, and are thus unlikely to be reproducible.

An ensemble study of a 10-residue peptide [33], designed to investigate the reproducibility of MD simulations, indeed displays the necessity for applying ensemble approaches to investigate peptide unfolding. In the study, 100 replicas were investigated with a simulation length of 3 µs each [33]. The study yet again concluded that single simulations are typically not reproducible [21]. Rather than using standard MD simulations, other approaches have been applied to sample the large conformational changes involved in peptide (un)folding, such as accelerated MD [64–66] and transition path sampling [55].

### 4.2. Ensemble-based sampling of restricted domains

In the study of a real biological system, it is practically not possible, and indeed not necessary, to sample extensively all regions in the configuration space. Only restricted regions of conformational space are important for the calculation of many properties of interest. The binding affinity, for example, is determined by the stable binding conformations. Accelerated MD approaches such as REST2 can help conformational sampling, but their propensity to drift from stable binding conformations degrades free energy predictions because of the lack of proper weighting factors for the conformations explored [61]. Precise predictions can be obtained when the most relevant conformations have been extensively sampled, without the need to explore a large conformational space.

The performance of short ensemble simulations for free energy predictions depends on the quality of initial binding poses and on the time scales for the efficient sampling of local conformations. The phrase ‘garbage in, garbage out’ is particularly pertinent when short simulations are used: a system will not be able to escape from a poor initial configuration within a limited simulation time, even if an ensemble is employed. The initial ensemble must be close to the most relevant region of the configuration space so that the relevant phase can be sampled extensively. With carefully prepared initial molecular systems, our studies show that the protocol of running 4-ns ensemble production runs works well for all the molecular systems we have investigated [21,25,26,51,59–61,77,101]; longer simulations only provide a marginal gain in the predictions [61], and may even have negative impact if the ligands drift away from their stable binding conformations [61]. There are certainly cases where longer simulation duration is required, either to improve the poor initial structures, to sample multiple binding conformations [77], or to obtain converged occupancy probabilities of water molecules at the binding sites [101,133]. A combination of conformational exploration and precise sampling, both based on ensemble simulation, may be required in these cases.

## 5. Distributed computing approaches to enhance sampling

In order to make a positive impact in industrial or clinical settings, computational predictions need to be made on time scales which can compete with or preferably dramatically outstrip the duration of experimental discovery and testing programmes. Today it is possible to achieve this by making use of the methods described here. The history of biomolecular simulation has been significantly influenced by the available computational power, the development of automated workflow tools, and the evolution of distributed computing approaches in recent years. In this section, we summarize the developments we and others have made in these areas.

### 5.1. Hardware approaches

Karplus recalled [134] the considerable courage required to perform the first MD simulation of a macromolecule of biological interest [135], due to the very limited and expensive computational resources available in the mid-1970s. Biomolecular simulation, along with other areas of computational science, have been benefiting from the rapid evolution of computing power. In the last five decades, the performance of microprocessors has improved exponentially, roughly following Moore's law which states that the number of transistors on a chip, a rough measure of processing power, doubles about every 2 years. Even though Moore's law has come to an end in recent years, the computational power is expected to keep improving for many years with the creative and/or specialized design of chips to accelerate specific crucial algorithms. There are also special-purpose supercomputers for MD simulations: Anton [131], for example, is a remarkable computer which can achieve simulation rates of microseconds per day for biosystems with millions of atoms.

Supercomputers use essentially the same microprocessors within nodes in far greater numbers than a desktop workstation, a fundamental difference arising from the speed of the interconnects linking nodes and cores together. While a typical early supercomputer from the 1980s only contained a few central processing units (CPUs), massively parallel designs since the 1990s have driven the architecture of supercomputers and connected a much greater number of microprocessors together via fast networking interconnects. Supercomputers today consist of hundreds of thousands to millions of such cores [83]. Indeed, cores as such are seldom referred to today; the basic units are the nodes, which typically contain tens to hundreds of cores and many also include accelerators.

The advent of GPU accelerators has resulted in more and more powerful specialized processing units designed for floating-point calculations. Initially used to accelerate the creation of images in a frame buffer, such processors are now in widespread use in high-performance computing and cloud platforms in so-called general-purpose GPUs. High-performance computing systems that feature both GPU accelerators and CPU chips within individual nodes allow hybrid applications to be developed that take advantage of the processing power GPUs offer, albeit at greater financial cost. MD codes developed to run on GPUs often show very significant performance improvements compared to CPU variants.

The explosion in the growth of computing power has led computational biologists to become prominent users of high-performance computing. Concomitantly, the rise of cloud computing has made accessing such resources at scale potentially trivial for researchers in academia and industry. The pay per use models promoted by clouds mean that users can pay just for the resources that they need to achieve their research objectives, without having to engage in expensive hardware procurement and operational costs, although this is a model which often works better in commercial contexts than within academic research. Notwithstanding these comments, at the level of compute intensity required to perform many free energy calculations, cloud computing can currently become prohibitive very rapidly on cost grounds.

### 5.2. Software approaches

To make the best use of available HPC resources, considerable effort has been devoted to improve the scalability of software and to develop adaptive and automated workflows. The scalability of MD codes on large numbers of cores and/or nodes is an important factor in determining the size and time scales of a problem which can be studied. Most of the MD codes used today have been designed or adapted to run on parallel computer systems. NAMD [136], for example, designed for high-performance simulation of large biomolecular systems, has been used to simulate systems consisting of tens of millions atoms [137], although any MD codes with long-range interactions, which are communication bound, will not scale effectively in a strong sense to reach required time scales [138]. OpenMM [139] and ACEMD (the latter now uses the OpenMM kernels) [140], designed and optimized for GPUs, are among the fastest MD codes in terms of single GPU board performance.

The application of molecular modelling techniques to real-world problems involves a complex workflow which is extremely tedious and error prone if performed manually, especially when ensemble computing approaches are embraced. In the case of pharmaceutical drug discovery for example, anywhere from hundreds to tens of thousands of compounds may need to be screened within a few days. The number of ensemble runs is of the same order of magnitude as the number of compounds. Managing the execution of simulations and collation of output data mandates the adoption of automation techniques to make the process tractable and reduce the time to solution. The scarcity of automated software tools is one major obstacle limiting the wider application of free energy approaches in real-world problems.

In recent years, a good deal of effort has been expended to develop workflows that simplify some or all of the process of free energy calculation using alchemical approaches such as TI and FEP, and/or endpoint approaches like MMPBSA, MMGBSA and LIE (table 1). The workflows include the entire steps to plan, set up, simulate, and analyse the final results in an automated manner. As mentioned earlier, FEP+ [73] is a patented free energy calculation suite from Schrödinger Inc., designed to automate the setup and analysis of FEP. It has been promoted primarily to major pharmaceutical companies. The Amber free energy workflow (FEW) [142] is a tool to set up alchemical and endpoint free energy calculations. FESetup [144] provides the setup of alchemical free energy and endpoint approaches for a few modelling packages including Amber, CHARMM, GROMACS, NAMD, Sire and OpenMM. The small-molecule topology generator (STaGE) [146] automatically generates GROMACS topologies using force fields such as Amber, CHARMM and OPLS, and sets them up for high-throughput free energy calculations, while pmx [145] within GROMACS provides an automated framework to provide hybrid protein/ligand structures and topologies for alchemical free energy calculations. Flare [147], implemented in Cresset's structure-based drug design suite, offers a graphical user interface to automate setup, simulation and analysis of free energy calculations via interfaces to the open source packages OpenMM, Sire, LOMAP, SOMD and BioSimSpace. We have developed our own free energy workflow called the binding affinity calculator (BAC) [141,148], designed to automate the end-to-end execution of ESMACS, TIES and ABFE calculations, and to handle ensemble calculations. A relative free energy calculation usually requires a hybrid topology and coordinate files to transfer one ligand to another. An automated algorithm is desired for planning and setting up free energy calculations between possible ligand pairs, and for generating required files. In most of the workflows mentioned here, this task is handled by the lead optimization mapper (LOMAP) [149] or other similar tools. The original LOMAP code was mainly based on commercial application programming interfaces (APIs) such as ones from Schrödinger and OpenEye. A new version of LOMAP has been developed, which is based on open APIs such as RDKit; this offers the scientific community a free tool to plan binding free energy calculations.

**Table 1.** Workflows to simplify free energy calculations. The workflows are designed to automate one or more of the multiple-step process of the calculation, with employment of endpoint and/or alchemical approaches in conjunction with specific force field(s) and MD engine(s). *✓*: function available; ✗: function not available.

name	automated steps			FE approaches		force field	MD engine	reference
	build	simulation	post-analyses	endpoint	alchemical			
FEP+	*✓*	*✓*	*✓*	✗	*✓*	OPLS	Desmond	[73]
BAC	*✓*	*✓*	*✓*	*✓*	*✓*	AMBER, CHARMM	NAMD, OpenMM, GROMACS	[141]
FEW	*✓*	*✓*	*✓*	*✓*	*✓*	AMBER	AMBER	[142]
YANK	*✓*	*✓*	*✓*	✗	*✓*	AMBER, CHARMM	OpenMM	[143]
FESetup	*✓*	✗	✗	✗	*✓*	AMBER	Sire, AMBER, GROMACS, CHARMM, NAMD, DL_POLY	[144]
pmx	*✓*	✗	*✓*	✗	*✓*	AMBER, CHARMM, OPLS	GROMACS	[145]
STaGE	*✓*	✗	✗	✗	*✓*	AMBER, CHARMM, OPLS	GROMACS	[146]
Flare	*✓*	*✓*	*✓*	✗	*✓*	AMBER	OpenMM	[147]

A complete free energy workflow comprises three distinct phases: (i) the preparation phase, (ii) the production phase and (iii) the analysis phase. The preparation phase is the step which all workflows focus on, in which the simulation-ready topology and coordinate files are constructed for a biomolecular system to be studied from its raw starting structure (usually in the form of a crystal structure in PDB format). They take parameter files for proteins, ligands and other components (water, ions, cofactors, etc.) as input with the specification of a desired force field. Some workflows also generate input configuration files compatible with chosen MD engines which are used to perform the equilibration and production simulations. After the successful execution of the simulation phase, the final step is to perform the statistical analysis on the output generated by the simulations or the post-processed data.

Most of the listed workflows are executed through a command line interface (CLI), with inputs being handled through shell scripts. An API is usually available, which allows a user to access the code through their own scripts for maximum flexibility and customization. As one might expect for a commercial product targeted at end-users in pharmaceutical companies, FEP+ has a well-designed graphical user interface to ensure user-friendly operation and high-quality visualization of input and output data. It uses a range of technical approaches to improve the accuracy and throughput of calculations, based on a replica-exchange method and GPU acceleration. However, as noted above, it is proprietary so users cannot access the code including the force field. As such, it fails to comply with the requirement of open and reproducible science and is thus harder to compare with other approaches. By contrast, a user friendly graphical interface for BAC, called ufBAC (see details below), has been developed in order to make it available to the widest range of users possible from academic to industrial and clinical. BAC has built-in ensemble-based simulation capabilities in order to ensure the accuracy and precision of reported results.

### 5.3. Distributed computing approaches

The significant computing resources required to deliver accurate binding predictions mean that we necessarily adopt a multitude of computing paradigms in order to perform investigations using BAC. To that end, we make use of large scale supercomputing class resources, as well as public cloud infrastructures including Azure, Amazon Web Services (AWS) and DNAnexus.

In the context of supercomputing class resources, we typically make use of RADICAL-Cybertools [150], developed by Rutgers University, a suite of tools that provide a common, consistent and scalable approach to high-performance and distributed computing. RADICAL-Cybertools consists of three fundamental components: (i) SAGA, an OGF community standard API for application-level jobs and data movement; (ii) RADICAL-Pilot (also known as BigJob), a tool that provides the ability to aggregate large number of tasks into a single-container job; (iii) EnsembleMD Toolkit, which builds upon RADICAL-Pilot as the execution layer, supports different patterns of ensemble-based computing, including replica exchange, workflows and simulation-analysis loops. These tools prove effective when wrapping complex workflows requiring very high core counts, and hence allow us to easily run replicas using the BAC Production component. RADICAL-Cybertools allows users to circumvent limitations put in place by supercomputing queuing systems, and run our applications in an efficient manner and ultra large scale including at the emerging exascale. A further feature of RADICAL-Cybertools allows adaptive applications to be created which alter the execution pattern based on the evolution of parameters in a defined set of simulations in order to promote computational efficiency.

**Figure 2.**
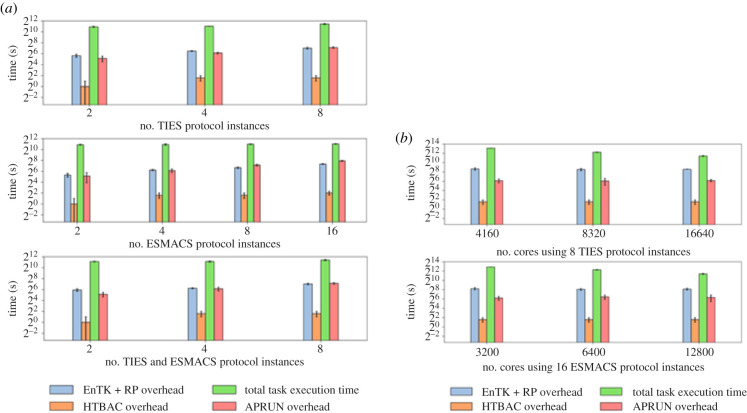
Weak and strong scaling of HT-BAC executed on BlueWaters, a Cray XE6/XK6 Supercomputer. (*a*) The ratio of number of protocol instances to resources is constant. Task execution time with HT-BAC, EnTK + RP, aprun overheads for TIES, ESMACS and a combination of TIES and ESMACS. (*b*) The number of protocol instances is fixed while the number of cores increases. Task execution time with HT-BAC, EnTK + RP, aprun overheads with TIES and ESMACS.

RADICAL-Cybertools has been used to develop a version of BAC called high throughput or HT-BAC, designed to maximize simulation throughput when running on such high-performance computing platforms. Computational studies [8] have found that HT-BAC exhibits near linear weak and strong scaling when running both ESMACS and TIES BAC simulations, as shown in figure 2. Furthermore, the adaptive computing capabilities of RADICAL-Cybertools are employed by HT-BAC when running TIES calculations to automate runtime decisions based on partial simulation data and redistribute resources at runtime to support dynamically generated simulations.

Deploying BAC to cloud resources has required us to adopt technologies that minimize the differences between platforms. The rise of cloud computing has been accompanied by the development of so-called ‘containerization’ technologies, which allow a whole operating system to be virtualized [151]. Effectively this means that, using primarily the Docker [125] tool, it is possible to create a fixed, standard and portable environment for applications, by wrapping them in Docker containers. (On HPC platforms, which operate a different security model, Singularity is a preferred container technology—see below.) This means that the different components of the BAC application can each be embedded in their own container, including all of the dependencies required to run them. Each containerized BAC component is stored in a container registry, and can easily be deployed to any infrastructure that supports Docker (and/or Singularity).

This process is made easier using Kubernetes, an open source orchestration system for the automated deployment, scaling and management of the containerized application. Kubernetes allows us to define virtual clusters on a cloud, which can then be used to run containerized applications. These clusters can then be scaled up or down in order to execute the required number of replicas. In the context of the BAC application, it provides us with an infrastructure-independent platform on which to execute application components. Kubernetes is available as a managed service on Azure, AWS and Google Cloud Platform, meaning that the containerized BAC application can be easily ported between the leading cloud platforms, and indeed to any platform that supports Kubernetes and Docker. A base Docker image contains the core of each application component, and this is then used to build Docker images for specific cloud platforms that contain the necessary code to move data around that platform for example.

As well as facilitating platform-independent cloud deployments, the use of containerization is becoming increasingly widespread on supercomputing class resources, using either Docker or Singularity [139] (which is compatible with Docker containers). This means that containerized BAC components can easily be deployed to compatible supercomputing resources, without users having to have recourse to system administrators to install and optimize application dependencies.

But the ability to easily and reliably deploy components of the application on cloud and supercomputing resources is not sufficient to allow users to trivially perform complex investigations using the software. To remedy this, we have developed ufBAC [148], a Web portal interface to the BAC, which allows a user to build models of molecule-compound binding, and execute and analyse multi-replica MD simulations using the model. ufBAC enables BAC to be run via a Software as a Service model, hiding from the user the complexities of the command line tools used to build models, execute them and analyse the results. ufBAC is intended to plug in to a range of computational back ends, from HPC resources provided by academic national research facilities to commercial platforms such as Microsoft Azure or AWS.

The purpose of the ufBAC system, and the portal in particular, is to make the process of running complicated simulation workflows that rely heavily on HPC as simple as possible, improving usability by moving the user away from the command line towards a user friendly cloud style application. The ufBAC Web portal follows a conventional design. The left-hand side of the interface contains a menu bar that allows the user to access the various features of the application. The top bar of the website displays user notifications (for example that a set of simulations has finished running). The main content panel gives access to the features of the application and allows users to control running simulations, create and execute new models and analyse data.

**Figure 3.**
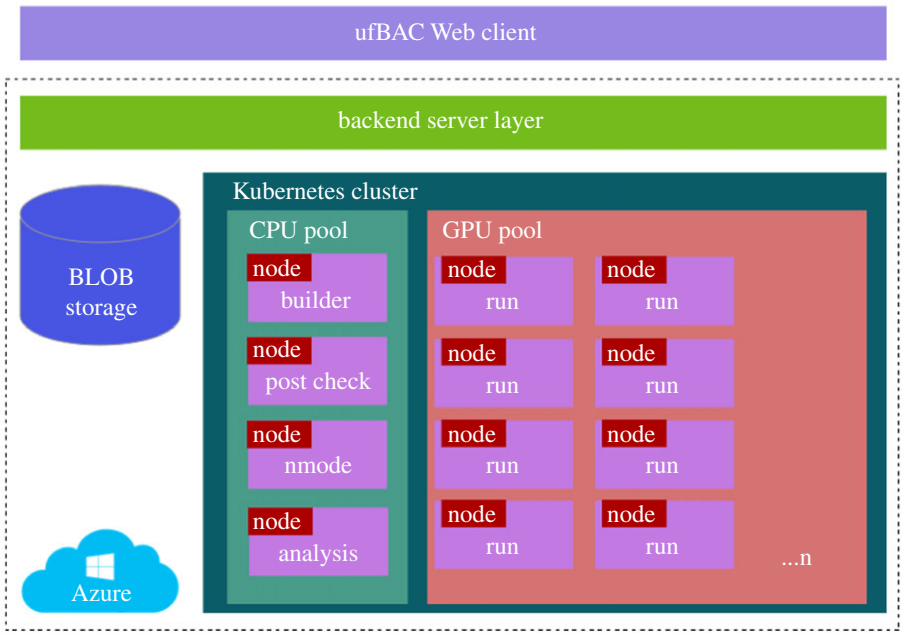
A deployment of the ufBAC application on the Azure cloud platform, using Kubernetes clusters and Docker containers. The main components of the application are encapsulated in Docker containers, the execution of which is managed by an application server layer. The user interacts with this layer via a Web portal.

A typical deployment of ufBAC, with BAC simulations performed on the Azure cloud using docker and Kubernetes, is outlined in figure 3. The application architecture comprises multiple layers. The first is the client layer, a user portal developed using Google Web Toolkit (GWT) [73]. The user interacts with the BAC system via their Web browser. The use of GWT provides a mechanism to develop high-performance, low overhead Web interfaces developed using Java which are compiled into separate JavaScript/HTML and Java byte code components, with the former running inside the user's browser (reducing server overheads) and interacting with the latter running inside a web application container such as Tomcat. It also means that new interfaces (designed for mobile devices, for example) can easily be constructed which make use of the common functionality provided by the server-side of the client.

The server layer comprises the server components of a set of RESTful Web services that allow the user to control the execution of the different BAC application components in the cloud. Services support user login, state management and collaboration. It interfaces with the Kubernetes cluster used to run the BAC application components. This cluster comprises two to three compute pools, made up of differing types of cloud nodes (for example, a single core pool for building and analysing jobs, a GPU pool for production simulation, and a multicore pool to run normal mode calculations). In addition, a cloud blob store is used to store file-based output from the different components of the BAC application, and to pass data between components.

## 6. Application of free energy calculations

As we have emphasized, the most effective and reliable computational route to the reproducible ranking of the binding affinity of ligands to proteins can be achieved using ensemble methods. The endpoint approaches usually impose no significant restriction on the nature of the drug–protein systems that can be studied, although careful thought/attention needs to be given to the setting up of the models in many instances, such as the positions of metal ions and binding site water molecules [133], the parametrizations for the ligands, and so on. A study of box size dependence in simulations opens a debate [152–154], which indeed highlights the importance of setting up systems for simulation correctly and, more importantly, applying ensemble approaches to get statistically significant results.

We should point out that because of the flexibility of endpoint approaches, they are well suited to the early stage of drug discovery, so-called hit-to-lead. The approach does not provide accurate ‘absolute’ free energies, because of the nature of the approximations used, such as the implicit solvent models in MMPBSA-based approaches and the linear reaction assumption in LIE. However, the ensemble simulation based approaches can yield precise and reproducible, hence reliable, binding affinity ranking predictions. The alchemical approaches are in principle both accurate and precise in their domain of applicability [74]. The use of ensembles allows for the modelled systems to vary within a large phase space [21,25] and thereby describes a population of all relevant models which provide probabilistic predictions about the system behaviour.

The approach has been applied in different areas, including structural determination of proteins by combined experimental and computational information [155], and for structure predictions of ligand–protein complexes using docking approaches [156]. For binding free energy calculations, the ensemble approach is increasingly widely employed as the most effective way forward. Williams-Noonan *et al.* [157] have summarized some case studies using alchemical approaches. Here, we review some of the free energy applications, using endpoint and/or alchemical approaches, to relatively large datasets that closely mimic a real-world drug development setting, and to a few clinically approved drugs binding to sequence-dependent target proteins in a more forward-looking approach for personalized medicine. As the ensemble approaches are only now beginning to make their way into real-world problems, the applications reviewed below are not limited to ensemble-based methods. It needs to be emphasized once again here that, due to the random nature of MD trajectories, one-off simulations do not have any reliability; only ensemble simulations enable one to draw statistically significant conclusions [21].

In direct collaboration with various leading pharmaceutical companies, we have tested the ensemble free energy approaches in realistic pharmaceutical settings [59,60]. The calculations were performed, initially blind, to investigate the ability of our ESMACS and TIES methods to reproduce the experimentally measured trends which were released to us by the pharmaceutical companies after our computational predictions were made. Very good correlations were obtained from both of the methods. In addition to the binding free energy, structural, energetic and dynamic information at the atomistic level is forthcoming from the simulations, which cannot be obtained experimentally. Such information not only explains experimental observations, it sheds light on how to make modifications in the laboratory to improve the ligand binding and/or ligand selectivity [59,60].

Wang *et al*. [73] published a study with a large number of compounds binding to eight proteins. A total number of 330 relative binding free energies were calculated using single trajectory-based FEP+. While most of the published free energy studies focused primarily on retrospective predictions, the study [73] also included two prospective projects, where some of the compounds had been synthesized based on the alchemical free energy calculations. While the description of the prospective study was brief without revealing the structures of the compounds simulated and synthesized in this study, more details have been presented in a similar prospective study of GPCRs [158]. Based on the computational predictions, four novel compounds were synthesized and experimentally tested, showing that simulations correctly predicted the binding affinities for two of them. FEP+ was also used for the calculation of relative binding free energies of fragment-sized compounds using several pharmaceutically relevant targets with 96 fragments [159]. The studies demonstrate that such alchemical approaches have the potential to guide the synthesis of potent compounds, to impact fragment-based affinity optimization and to assist rational drug design projects.

Another less explored potential application of free energy prediction approaches is in the area of personalized medicine [82]. Due *inter alia* to the acquisition of drug resistance by individuals, it is often necessary to tailor the medication of a person according to his/her genetic configuration. In such cases, an accurate ranking of the available drugs based on their binding affinities when bound to different mutants of the target protein is the prerequisite. We studied the efficacy of two inhibitors to wild-type and mutant fibroblast growth factor receptor 1 (FGFR1) using ensemble simulations [75]. FGFR1 is a recognized therapeutic target in cancer. The binding affinities we predicted were confirmed by later-revealed biochemical measurements from our laboratory-based colleagues. The accuracy of the results displays the potential for the method to be applied in personalized medicine. Hauser *et al*. [160] recently published a study to predict how protein mutations modulate inhibitor affinities to Abl kinase. Classification of mutations as resistant or susceptible was predicted with a reasonable accuracy for eight FDA-approved drugs across 144 clinically identified mutations. Fowler *et al*. [161] demonstrated that ensemble alchemical approaches generated quantitatively accurate free energies, and were able to estimate the specificity and sensitivity of mutations in *Staphylococcus aureus* dihydrofolate reductase.

Point mutations in proteins can occur either inside (‘local’ mutants) or away from (‘remote’ mutants) the binding pocket. It is worth mentioning here that alchemical free energy methods may not be able to correctly predict binding affinities in the case of remote mutants—the ones spatially a long way from the binding site—on typical wall clock time scales. Even using accelerated sampling techniques like REST2 cannot guarantee to improve the accuracy of such predictions [77] for reasons we alluded to earlier. Indeed an apparent underestimation from a recent study [78] calls attention to the need of further studies to validate the REST2 method in free energy calculations. A critical analysis of the application of alchemical free energy methods on protein mutations has been performed recently in our group [61]. There, we provide insights underpinning the impact of the gatekeeper mutation (a ‘local’ mutant) of FGFR3 on drug efficacy using ensemble approaches with the REST2 method. We focus on the UQ in these methods and, using that metric, we are able to compare the performance of different software and hardware for the calculation of the same free energy changes and show that, using ensemble-based methods, one can achieve reproducible results.

## 7. Conclusion

Drug–receptor binding is of key importance in determining drug efficacy and safety. The molecular determinants of binding affinity, compared with those of binding kinetics, are well understood. Binding free energy calculations are, therefore, expected to provide valuable contributions in real-world problems such as in rational drug design as a virtual screening and optimization tool, and in personalized medicine as a component of clinical decision support systems [82,162]. Numerous approaches and software tools have been developed for the purpose. Despite the applicability of the technology being well established, the methods have not thus far become standard virtual screening tools within the pharmaceutical industry, still less for decision support systems in clinical practice. The latter is a very new application of free energy calculations, being discussed only in recent years [18,26,82]. What aspects are limiting its applicability at present, and how can significant progress be made in the future?

While recent developments of the approaches have resulted in major improvements over what was available just a few years ago, there are still limitations in applying them within industry. These limitations include: the accuracy of the predictions, the challenge of handling truly diverse datasets, the general usability, as well as the computational power and financial cost required. While all of these limitations have been alleviated by the recent advances in software, middleware and hardware, novel approaches are required to further improve the accuracy of the predictions. Diverse datasets, including the incorporation of a variety of crystal structures, can succumb to careful preparation and analysis [40], including careful use of and standardization of the software, choice of force fields and assignment of partial charges, as well as selection of bound water molecules to include in the computations. It should be noted that often the sought correlation of the computed free energy is done against something not rigorously related to it, such as IC_50_, and insufficient attention has been paid to the errors in those experimental measurements [14,51,73,163]. To date only a limited number of studies have been reported which compare free energy calculations from different MD codes and force fields due to technical difficulties of comparing with every code. Studies have shown that consistent results can be obtained across different MD engines [41,61,78]. Our recent investigations using three MD engines and two force fields show that the influences of force fields and MD codes on results are often quite small if ensemble methods are used as the basis for such comparisons [78].

In different scenarios, the cost–performance ratio can be in favour of cloud environment or on premise HPC facilities. While the former is usually preferred over the latter for small applications, traditional HPC facilities are what large scale tightly coupled calculations usually demand [164]. The equivalent instance hours required for MD simulations usually make the cloud applications prohibitive for many users, certainly in the academic community where investigators lack the supplies of cash required to meet such bills, but also in many companies; moreover, use of ensemble simulation substantially increases the costs. These factors make industrial users dither over committing to clouds when they could buy on premise HPC hardware. The current situation is that many users still prefer to own their own computers to avoid issues associated with the security of data, as well as cost. Nonetheless, the concepts of containerization and virtual machines are important advances brought about by the cloud computing paradigm which have entered mainstream HPC too in recent times [151]. In the long term, it is likely that both will be used, perhaps with bursting out to off-premises clouds when the workload cannot be handled internally. As mentioned above in the context of ML, reducing computational cost and time to solution is one of the motivations for invoking ML methods. However, UQ applied to ML predictions is in its infancy, being less mature than that we apply to MD simulation data.

Using ensemble methods, the errors in predictions can be systematically controlled, amenable to further reduction by increasing the number of replicas in an ensemble and by extending the length of simulations. Ensemble approaches are scalable, allowing thousands of binding affinities to be calculated per day, depending only on the computing resources available. Using ESMACS, for example, a single ligand–protein target can be assessed in 1–2 h using GPUs [61]. TIES is more computationally expensive, but predicts changes in free energy between pairs of structurally closely related ligand–protein systems. A binding free energy difference can be calculated in 2 h with modern codes running on accelerators, using HPC or cloud resource offering the latest GPU technology. Automated workflows are essential, which significantly increases the usability of the methods, while scale out to very large supercomputers makes it possible to deliver actionable predictions. Indeed, as evidenced by our Giant Workflow [165] on the entirety of Phase 1 and Phase 2 of SuperMUC, two linked supercomputers at the Leibniz Supercomputing Centre in Garching near Munich, with a combined total of about 245 000 cores where we achieved a sustained performance of about seven petaflops, it is now possible for us to produce high-quality binding affinity predictions for of the order of several hundred target proteins within a day or so with suitable computing resources. It is foreseeable that in the near future, rapid and accurate free energy prediction at high throughput will assist medicinal chemists in planning and directing compound synthesis in a routine manner.

Ensemble simulation-based free energy prediction approaches provide a route to predict relevant drug–protein binding affinities and hence are directly applicable in rational drug development and personalized medicine. They yield precise and reproducible, hence reliable, binding affinity ranking predictions. They should provide a major boost to rational drug development in the pharmaceutical industry, and to personalized medicine in clinical practice. To be sure, there are still obstacles remaining, for example, in modelling charge-changing mutations and sampling relevant phase space when large conformational transitions occur. For mutations involving net charge changes, the long-range electrostatic interactions need to be incorporated properly [106]. Substantial conformational changes, which could be triggered by mutations involving charge changes or large size changes [61], are still a major issue for the convergence of free energy predictions. The double annihilation approach in ABFE calculations will almost always engender large conformational changes; the accuracy and convergence of the predictions need to be improved for the method to be used for a wider set of biologically interesting problems. The calculations are very computationally intensive, which is related to the requirement of sampling a representative conformational ensemble so as to get accurate and precise predictions. More efficient conformational search methods, which can access all of the energetically important conformational states, will significantly enhance the value of MD-based free energy calculations. With the improving theories and models, the increasing availability of automated tools and access to yet more powerful computing resources, binding free energy calculations are coming of age as a computational tool for the pharmaceutical industry and, in the longer term, to clinicians for drug selection in the context of personalized medicine.

## Data accessibility

This article has no additional data.
